# The Value of Four Anthropometric Indicators for Identifying Left Ventricular Hypertrophy in Chinese Hypertensive Patients

**DOI:** 10.1155/2022/6842825

**Published:** 2022-05-17

**Authors:** Bokai Cheng, Nan Lu, Ge Song, Jiaojiao Qiu, Jing Dong, Shuang Cai, Yongkang Su, Jin Sun, Anhang Zhang, Qiligeer Bao, Man Li, Shouyuan Ma, Yan Zhang, Ping Zhu, Shuxia Wang

**Affiliations:** ^1^Medical School of Chinese PLA, Chinese PLA General Hospital, Beijing, China; ^2^Department of Geriatrics, The Second Medical Center and National Clinical Research Center for Geriatric Diseases, Chinese PLA General Hospital, Beijing, China; ^3^Health Division of Guard Bureau, Joint Staff of the Central Military Commission, Beijing, China; ^4^Department of Geriatric Cardiology, The Second Medical Center, Chinese PLA General Hospital, Beijing, China; ^5^Department of Outpatient, The First Medical Center, Chinese PLA General Hospital, Beijing, China

## Abstract

Left ventricular hypertrophy (LVH) has been classified separately as an independent risk factor for hypertension. However, comparisons between different body size indices and left ventricular hypertrophy (LVH) in hypertensive populations have not been reported yet. In this study, we enrolled 4,639 hypertensive patients from rural China. Anthropometric indices and cardiovascular disease risk factor variables were measured and analyzed using Spearman's correlation, logistic regression, and receiver operating characteristic (ROC) curve analyses. Patients in the highest quartile of body size indices were more likely to have left ventricular hypertrophy than those in the lowest quartile; these indices were BMI (adjusted OR: 3.55, 95% CI: 2.90; 4.35), WC (adjusted OR: 2.23, 95% CI: 1.84; 2.70), WHR (adjusted OR: 1.44, 95% CI: 1.18; 1.75), and WHtR (adjusted OR: 3.23, 95% CI: 2.62; 3.99). The areas under the ROC curves of BMI (AUC: 0.628, 95% CI: 0.612; 0.644), WHtR (AUC: 0.628, 95% CI: 0.560; 0.593), WHR (AUC: 0.530, 95% CI: 0.513; 0.547), and WC (AUC: 0.576, 95% CI: 0.513; 0.547) were all above 0.5, which indicated that the four anthropometric indicators may be associated with LVH. The four anthropometric indicators of obesity were identified as risk factors for LVH. Weight control might help reduce the risk of left ventricular hypertrophy.

## 1. Introduction

The rates of overweight and obesity have increased rapidly in the last four decades, and obesity has become a major public health issue in China [1]. Obesity is one of the most critical risk factors for atherosclerotic cardiovascular disease, type 2 diabetes, and other metabolic diseases, leading to the loss of nearly 20 years of life [2–4]. Weight loss helps improve ventricular remodeling and heart failure [5]. The metabolic complications of obesity are more closely related to visceral obesity than overall obesity [6]. Compared to subcutaneous fat, visceral fat has solid endocrine activity and its secretions affect the cardiovascular system [7].

Imaging tests such as computed tomography [8] and magnetic resonance imaging [9] accurately identify obesity; however, they are not widely promoted due to cost and operation restrictions. Therefore, anthropometric methods are more practical as they are easy, repeatable, and convenient. BMI is a common standard for measuring obesity. However, it does not distinguish abdominal obesity from other types of obesity [10, 11]. The waist circumference (WC), waist-to-hip ratio (WHR), and waist-to-height ratio (WHtR) are all considered as indexes of abdominal obesity [12, 13].

LVH results from the combined effect of currently known cardiovascular risk factors, such as age, blood pressure, and obesity; therefore, it is considered a surrogate indicator of cardiovascular risk factors [14]. LVH significantly increases the risk of ischemic heart disease, heart failure, and stroke, especially in hypertensive populations [15–17]. Therefore, it is essential to study the predictors of left ventricular hypertrophy.

To our knowledge, there have been few studies on the relationship between anthropometric markers and LVH, especially in community-based hypertensive people. We conducted this population-based cross-sectional study to assess the ability of different anthropometric indicators to identify individuals with left ventricular hypertrophy in a rural hypertensive population in central China.

## 2. Materials and Methods

### 2.1. Population

This cross-sectional survey was conducted in rural communities in Anyang City, Henan Province, from 2004 to 2005. A multistage cluster sampling method was used to recruit members of rural communities aged 40–75 years as representative samples. A total of 13,444 people (5,270 males and 8,174 females) participated in the survey. Of the 5,421 hypertensive patients, 4,805 (89%) had measured LV mass and 166 participants were excluded due to the unavailability of anthropometric data. Finally, the clinical and echocardiographic data of 4,639 patients were included.

The ethical review of this study was conducted by Fuwai Hospital and local hospitals. The subjects defined the nationality as the Han nationality.

### 2.2. Measurements

Participants' weights and heights were accurate to 0.1 kg and 0.1 cm, respectively. These parameters were measured with the participants putting on light clothing and bare shoes. We measured the waist circumference (with an accuracy of 0.1 cm) in standing subjects using a soft tape midway between the lowest rib and the iliac crest. We measured the hipline circumference using the location of the hip's greatest circumference with an accuracy of 0.1 cm. Blood pressure was measured by a professional using a standard medical mercury sphygmomanometer with an appropriate cuff selected according to the circumference of the participant's right arm. All participants were asked to avoid stimulating beverages and strenuous exercise prior to the blood pressure measurement. Three measurements were taken in a seated position after at least five minutes of rest, at least 30 seconds apart, and three readings were recorded.

### 2.3. Echocardiographic Method

Transthoracic echocardiography was performed according to standard protocols [18], including *M* mode, two-dimensional (2D), and color Doppler recordings from parasternal long- and short-axis windows and 2D and color Doppler evaluation from apical windows, using HP 5500 (Phillips Medical System, Boston, MA, USA) or HDI 3000 (ATL, Bothell, WA, USA) for yielding 2-, 3-, and 4-chamber images. The transducer frequency was 2.5–3.5 MHz. According to the recommendations of the American Society of Echocardiography, the internal diameter of the left ventricle, the thickness (IVST), and the posterior wall (PWT) were measured at end-diastole and end-systole. Two echocardiographers with at least two years of experience supervised the echocardiography. Before the study, technicians were trained in the echocardiography program of the Institute of Cardiology of the Chinese Academy of Medical Sciences.

### 2.4. Calculation of Derived Variables

The left ventricular mass (LVM) was calculated using the following equation: LVM (*g*) = 0.81 (1.04 [LVIDD + IVSD + PWT])^3^ − (LVIDD)^3^ + 0.06 [19]. The left ventricular mass index (LVMI) [20] was calculated by dividing the LVM by the Height^2.7^. The body mass index [21] (BMI) was calculated using the following formula: weight/height^2^. The waist-to-hip ratio (WHR) was calculated as follows: waist circumference (WC)/hipline circumference. The waist-to-height ratio (WHtR) was calculated as follows: waist circumference (WC)/height.

### 2.5. Definitions

LVH was defined as the LV mass index for height^2.7^ >46.7 g/m^2.7^ in women and >49.2 g/m^2.7^ in men [22]. The cut-off values for men and women were approximately 24 kg/m^2^ and 23 kg/m^2^ for BMI, 85 cm and 75 cm for WC, 0.50 and 0.48 for WHtR, and 0.90 and 0.85 for WHR, respectively [23, 24]. Hypertension was defined as a diastolic blood pressure of ≥90 mm·Hg or a systolic blood pressure of ≥140 mm·Hg, having been diagnosed with hypertension by a physician or currently taking hypertensive medication (WHO in 1999) [25].

### 2.6. Statistical Analysis

Continuous variables were presented as mean ± standard deviation, and categorical variables were expressed as frequencies and percentages. To analyze the differences between the LVH group and the non-LVH group, an independent-sample *t*-test was used for continuous variables and the chi-square test was used for categorical variables. Pearson's correlation coefficient was used to evaluate the correlation of BMI, WHtR, WHR, and WC with LVMI, LVM, and RWT. Logit model analysis was used to calculate ORs and 95% CIs. We assessed the relationship between different anthropometric indexes and LVH using a binary logit model and multivariate analysis. When the anthropometric index was applied as a continuous variable in the binary logit model, the WHtR was one-tenth of the original value. The BMI, WC, WHR, and WHtR values were divided into quartiles (Q1: <25%, Q2: ∼25%, Q3: ∼50%, and Q4: ∼75%). The relationship between each category and LVH was then assessed using a binary logit model and multivariate analysis. We used the receiver operating characteristic (ROC) curve analysis to evaluate the effect of four obesity indicators in predicting LVH and calculated the area under the curve. Statistical significance was assumed at *P* < 0.05. All statistical analyses were performed with SPSS 26.0 for Windows (SPSS Inc., Chicago, IL, USA).

## 3. Results

### 3.1. Clinical and Echocardiographic Characteristics of the Study Participants

Data on the clinical characteristics of the included participants are shown in Table 1. Subjects with LVH were older than those without it. Compared to patients without LVH, patients with LVH had higher SBP, DBP, TG, LDL-C, WC, HC, BMI, WHtR, and lower HDL-C levels, and the above differences were statistically significant. Interestingly, the difference in the WHR between the two groups was not statistically significant. Concerning echocardiographic parameters, the average levels of LVIDD, IVSd, PWT, and RWT in the LVH group were significantly higher than those in the non-LVH group.

### 3.2. Pearson's Correlation Coefficient of Anthropometric Measures and the LVH Index

The BMI, WHtR, WHR, and WC were associated with LVM as determined by the Pearson rank test, and the correlation coefficients were 0.23, 0.14, 0.08, and 0.27, respectively. The BMI, WHtR, and WC were also associated with the LVMI (except for the WHR) as determined by the Pearson rank test, and the correlation coefficients were 0.26, 0.26, and 0.17, respectively. WHtR was associated with RWT as determined by Pearson's rank test alone (Table 2).

### 3.3. Relationship between Anthropometric Indexes and Left Ventricular Hypertrophy

The results of the logistic regression analysis of anthropometric indices suggested that BMI (OR: 1.14, 95% CI: 1.12–1.16), WC (OR: 1.03, 95% CI: 1.02–1.04), and WHtR (OR: 2.14, 95% CI: 1.193–2.36), when used as continuous variables, are risk factors for left ventricular hypertrophy. For every 0.1 increase in WHtR, there is a 1.14-fold increase in the risk of left ventricular hypertrophy. In addition, BMI, WC, and WHtR remained risk factors for LVH after adjusting for age, sex, systolic blood pressure, diastolic blood pressure, serum glucose, triglyceride, low-density lipoprotein cholesterol, blood urea nitrogen, and serum uric acid levels (Table 3).

Patients in the highest quartile were more likely to have left ventricular hypertrophy than those in the lowest quartile for the four anthropometric indices, i.e., BMI (adjusted OR: 3.55, 95% CI: 2.90–4.35), WC (adjusted OR: 2.23, 95% CI: 1.84–2.70), WHR (adjusted OR: 1.44, 95% CI: 1.18–1.75), and WHtR (adjusted OR: 3.23, 95% CI: 2.62–3.99) (Table 4).

Furthermore, we stratified the analysis into different sex and age groups. The results showed that the effects of the highest quartile of all anthropometric indices with an increased risk of LVH did not change in the different sex and age groups (Table 5).

### 3.4. ROC of Each Anthropometric Index

**Figure 1:**
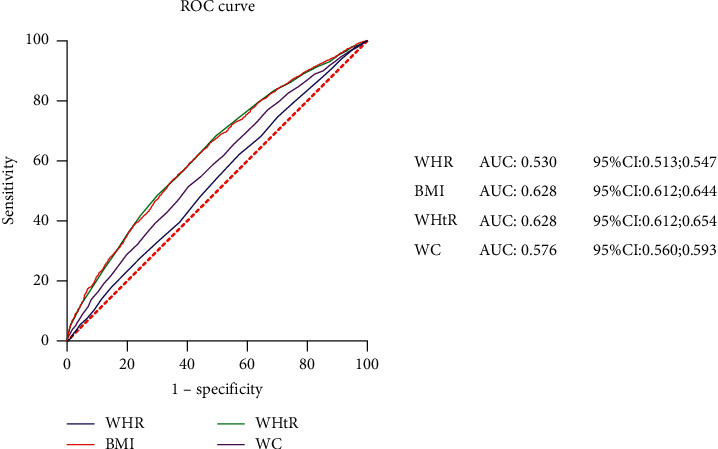
The ROC curve and area under the curve of BMI, WC, WHR, and WHtR.

The ROC curve and the area under the curve for BMI (AUC: 0.628), WC (AUC: 0.576), WHR (AUC: 0.530), and WHtR (AUC: 0.628) were all above 0.5, which indicated that the four anthropometric indicators could be associated with LVH (Figure 1).

## 4. Discussion

Our study indicated that the four anthropometric measures of obesity were risk factors for left ventricular hypertrophy. Abdominal obesity indexes such as WHtR, WC, and WHR were not superior risk factors to traditional methods such as BMI. The findings of our study are inconsistent with previous literature reports; for example, Ashwell et al. reported that WHtR was more advantageous than WC and BMI in measuring cardiometabolic risk in both sexes [12]. Wang et al. also reported that WHtR performed similarly or even better than BMI and WC among Chinese children [26]. We speculate that this difference may be due to the following reasons: Firstly, previous studies were mainly conducted in general community populations, while our study population was mainly composed of hypertensive patients in rural communities. Secondly, the local people have less medical knowledge; hence, the rates of therapy and hypertension control are lower [17, 27]. Thirdly, this population prefers a high-salt and high-fat diet, and among dyslipidemia patients, the proportion of patients with awareness, treatment, and control of the condition is relatively lower [28]. These factors, acting synergistically with obesity and hypertension, contribute to the development of left ventricular hypertrophy [20].

The relationship between obesity and left ventricular hypertrophy has not been elucidated, and it is currently thought that this relationship could be that obesity leads to altered hemodynamics, increased cardiac output and volume load, reduced left ventricular compliance, and, ultimately, ventricular remodeling [29–31]. Obesity also contributes to other cardiovascular risk factors such as dyslipidemia, HTN, glucose intolerance, inflammatory states, and obstructive sleep apnea [32]. Obesity can activate the renin-angiotensin-aldosterone system [33–35], leading to the retention of water and sodium, which increases the cardiac afterload and results in ventricular remodeling. Adipose tissue can secrete a hormone called leptin [36], and overweight and obesity can result in leptin resistance. In the mouse model, it was found that leptin could increase the activity of extracellular matrix metalloproteinases, promote interstitial fibrosis, participate in inflammatory reactions, induce the production of reactive oxygen species, and promote LVH [37].

Weight loss plays a vital role in reversing left ventricular hypertrophy; however, the exact mechanism by which this occurs remains unclear. Algahim et al. found a significant regression in LVM and LVMI after bariatric surgery and suggested that this change is modulated by neurohumoral factors that may improve long-term survival [38]. Without significant weight loss, the reversal of left ventricular remodeling is also generally not achieved by blood pressure control alone [5].

The diagnosis of hypertension is currently controversial. Our study was conducted from 2004 to 2005, and the diagnostic criteria for hypertension were consistent with those established by the WHO at that time. Currently, the diagnosis of hypertension in China is still based on a blood pressure of ≥140/90 mmHg [39–41]. The 2017 American College of Cardiology (ACC)/American Heart Association (AHA) guidelines for hypertension reduced the diagnostic threshold for hypertension to 130/80 mmHg. It is convenient for early diagnosis and intervention in the case of hypertension and reduces the occurrence of adverse events caused by the condition. However, hypertension is a global public health problem, and strict diagnostic criteria will also increase medical output and even cause other social problems [42].

However, this study had some limitations. First, more than 15% of eligible patients were excluded due to the unavailability of ultrasound and anthropometric data. We conducted a single-sample *t*-test. Gender assessment was performed using the chi-square test. The results showed that the baseline data, except for ultrasound and anthropometric data, were not significantly different from those of the general population, indicating that the sample can take the table as a whole (Supplementary Table 1). Then, we made a best-worst case analysis (assuming that the members of the population who were not followed up were suffering from either obesity and LVH (best case) or obesity without LVH (worst case); the anthropometric indicators were taken at extremes). The results showed that, in extreme cases, there is indeed an impact on the results (Supplementary Table 2). Second, this study was conducted in a Chinese Han hypertensive population, and the study sample was not fully representative of the general Asian population. Third, other factors known to influence the development of left ventricular hypertrophy, such as dietary habits and contributive family history, were not considered. Fourth, this was a cross-sectional study, and the results do not conclusively demonstrate a causal relationship between the various anthropometric indices and left ventricular hypertrophy. More prospective studies are needed to confirm the findings of this study. Finally, because of the cross-sectional study design, we could not differentiate between the effects of hypertension and those of obesity on LVH.

## 5. Conclusions

Four anthropometric indicators of obesity are risk factors for left ventricular hypertrophy. Weight control might help reduce the risk of left ventricular hypertrophy.

## Figures and Tables

**Table 1:** Comparison of indexes between the LVH group and the non-LVH group.

Clinical characteristics	LVH (1923)	Non-LVH (2716)	*P* value
Age (years)	59.1 ± 8.3	57.4 ± 8.8	*P* < 0.01
Male (%)	29.5%	36.6%	*P* < 0.01
SBP (mmHg)	168.7 ± 25.5	160.0 ± 22.9	*P* < 0.01
DBP (mmHg)	98.2 ± 13.6	96.3 ± 11.6	*P* < 0.01
HR	72.3 ± 12.0	72.9 ± 12.4	0.115
Height (m)	1.56 ± 0.076	1.59 ± 0.081	*P* < 0.01
Weight (kg)	66.57 ± 17.95	64.67 ± 10.93	*P* < 0.01
GLU (mmol/L)	5.51 ± 1.65	5.60 ± 1.72	0.84
TC (mmol/L)	5.53 ± 1.09	5.55 ± 1.10	0.59
TG (mmol/L)	1.76 ± 1.31	1.64 ± 1.19	*P* < 0.01
HDL-C (mmol/L)	1.53 ± 0.33	1.57 ± 0.35	*P* < 0.01
LDL-C (mmol/L)	3.16 ± 0.87	3.15 ± 0.84	0.60
BUN (mmol/L)	5.54 ± 1.85	5.41 ± 1.78	*P* < 0.05
Anthropometric indexes			
Waist circumference (cm)	86.69 ± 9.93	84.98 ± 9.54	*P* < 0.01
Hipline circumference (cm)	99.41 ± 11.97	97.49 ± 9.99	*P* < 0.01
Waist-to-height ratio (WHtR)	0.55 ± 0.08	0.53 ± 0.07	*P* < 0.01
Waist-to-hip ratio (WHR)	0.87 ± 0.06	0.87 ± 0.15	0.19
Body mass index (BMI)	27.22 ± 4.17	25.48 ± 3.46	*P* < 0.01
UCG indices			
LVIDD (mm)	48.21 ± 4.71	43.55 ± 4.45	*P* < 0.01
PWT (mm)	10.54 ± 1.27	9.14 ± 1.09	*P* < 0.01
IVSd (mm)	10.90 ± 1.48	9.33 ± 1.34	*P* < 0.01
RWT (mm)	0.45 ± 0.08	0.42 ± 0.07	*P* < 0.01

SBP: systolic blood pressure; DBP: diastolic blood pressure; HDL: high-density lipoprotein; LDL: low-density lipoprotein; TG: triglycerides; TC: serum total cholesterol; BUN: blood urea nitrogen; LVMI: left ventricular mass index; RWT: relative wall thickness; IVSd: end-diastolic interventricular septal thickness; LVIDD: end-diastolic internal dimension.

**Table 2:** Pearson's correlation coefficient of anthropometric measures and the LVH index.

Variables	BMI	WHtR	WHR	WC
LVMI (LVM/height^2.7^)	0.26^*∗*^	0.26^*∗*^	0.03	0.17^*∗*^
LVM	0.23^*∗*^	0.14^*∗*^	0.08^*∗*^	0.27^*∗*^
RWT	0.003	0.05^*∗*^	0.03	0.02

^∗^ *P* < 0.01.

**Table 3:** Relationship between anthropometric indexes and left ventricular hypertrophy.

Variables	Unadjusted OR	*P*	Adjusted OR	*P*
WHtR (0.1)	2.14 (1.193–2.36)	<0.01	2.18 (1.94–2.45)	<0.01
BMI	1.14 (1.12–1.16)	<0.01	1.15 (1.13–1.18)	<0.01
WHR	1.33 (0.73–2.42)	0.35	1.34 (0.71–2.51)	0.37
WC	1.03 (1.02–1.04)	<0.01	1.04 (1.03–1.042))	<0.01

OR = odds ratio, adjusted for age, sex, systolic blood pressure, diastolic blood pressure, serum glucose, triglyceride, low-density lipoprotein cholesterol, blood urea nitrogen, and serum uric acid. WHtR: waist-to-height ratio; WC: waist circumference; BMI: body mass index; WHR: waist-to-hip ratio.

**Table 4:** OR (95% CI) for LVH by the quartiles of BMI, WC, WHR, and WHtR.

Index	Q1	*P* value	Q2	*P* value	Q3	*P* value	Q4	*P* value
BMI	≤23.64		23.64∼26.02		26.03∼28.50		≥28.51	
Crude	Reference		1.54 (1.29; 1.83)	＜0.01	2.12 (1.79; 2.52)	＜0.01	3.23 (2.71; 3.84)	＜0.01
Model 1	Reference		1.70 (1.41; 2.05)	＜0.01	2.34 (1.95; 2.81)	＜0.01	3.62 (3.01; 4.35)	＜0.01
Model 2	Reference		1.72 (1.42; 2.10)	＜0.01	2.40 (2.00; 2.92)	＜0.01	3.55 (2.90; 4.35)	＜0.01
WC	≤79		80∼86		87∼92		≥93	
Crude	Reference		1.37 (1.16; 1.61)	＜0.01	1.65 (1.38; 1.98)	＜0.01	2.00 (1.70; 2.36)	＜0.01
Model 1	Reference		1.45 (1.22; 1.72)	＜0.01	1.81 (1.51; 2.23)	＜0.01	2.23 (1.87; 2.65)	＜0.01
Model 2	Reference		1.43 (1.19; 1.72)	＜0.01	1.75 (1.42; 2.15)	＜0.01	2.23 (1.84; 2.70)	＜0.01
WHtR	≤0.5		0.51∼0.54		0.55∼0.58		≥0.59	
Crude	Reference		1.56 (1.31; 1.86)	＜0.01	2.14 (1.79; 2.56)	＜0.01	3.37 (2.82; 4.03)	＜0.01
Model 1	Reference		1.57 (1.31; 1.88)	＜0.01	2.11 (1.75; 2.55)	＜0.01	3.21 (2.65; 3.89)	＜0.01
Model 2	Reference		1.64 (1.32; 1.95)	＜0.01	2.23 (1.82; 2.73)	＜0.01	3.23 (2.62; 3.99)	＜0.01
WHR	≤0.83		0.84∼0.87		0.88∼0.90		≥0.91	
Crude	Reference		1.21 (1.03; 1.41)	＜0.05	1.22 (1.02; 1.47)	＜0.05	1.34 (1.13; 1.59)	＜0.05
Model 1	Reference		1.30 (1.10; 1.52)	＜0.05	1.33 (1.10; 1.60)	＜0.05	1.59 (1.33; 1.92)	＜0.05
Model 2	Reference		1.21 (1.02; 1.44)	＜0.05	1.22 (1.00; 1.49)	＜0.05	1.44 (1.18; 1.75)	＜0.05

Model 1: adjusted for age and sex. Model 2: adjusted for age, sex, systolic blood pressure, diastolic blood pressure, serum glucose, triglyceride, low-density lipoprotein cholesterol, blood urea nitrogen, and serum uric acid.

**Table 5:** OR (95% CI) of LVH for the highest quartile versus the lowest quartile of BMI, WC, WHR, and WHtR.

	BMI		WC		WHtR		WHR	
	OR (95% CI)	*P* value	OR (95% CI)	*P* value	OR (95% CI)	*P* value	OR (95% CI)	*P* value
Sex								
Male	4.41 (3.05; 6.36)	<0.01	1.65 (1.25; 2.17)	<0.01	3.08 (2.11; 4.50)	<0.01	1.78 (1.23; 2.57)	<0.05
Female	3.53 (2.78; 4.49)	<0.01	2.27 (1.85; 2.79)	<0.01	3.70 (2.86; 4.78)	<0.01	1.45 (1.14; 1.83)	<0.05
Years								
<55	3.63 (2.56; 5.15)	<0.05	2.06 (1.49; 2.86)	<0.05	3.65 (2.56; 5.22)	<0.01	1.30 (0.94; 1.81)	0.118
≥55	3.57 (2.80; 4.58)	<0.05	2.24 (1.77; 2.83)	<0.01	3.05 (2.36; 3.93)	<0.01	1.67 (1.31; 2.11)	<0.01

OR = odds ratio, adjusted for age, sex, systolic blood pressure, diastolic blood pressure, serum glucose, triglyceride, low-density lipoprotein cholesterol, blood urea nitrogen, and serum uric acid.

## Data Availability

The datasets used in the study are available from the corresponding author upon reasonable request.

## Abbreviations

LVH: Left ventricular hypertrophy
LVMI: Left ventricular mass index
RWT: Relative wall thickness
IVSd: End-diastolic interventricular septal thickness
LVIDD: End-diastolic internal dimension
BMI: Body mass index
SBP: Systolic blood pressure
DBP: Diastolic blood pressure
WHtR: Waist-to-height ratio
WC: Waist circumference
WHR: Waist-to-hip ratio
HC: Hipline circumference
HDL: High-density lipoprotein
LDL: Low-density lipoprotein
TG: Triglycerides
TC: Serum total cholesterol.

## Supplementary Materials

Supplementary Materials Supplementary Table 1: totality data vs sample data. Supplementary Table 2: best-worst case analysis.
