# Supplementary material for: The Value of Four Anthropometric Indicators for Identifying Left Ventricular Hypertrophy in Chinese Hypertensive Patients
Source: Int J Hypertens. 2022 May 17;2022:6842825. doi: 10.1155/2022/6842825 (PMC9129951; doi:10.1155/2022/6842825)
Supplement: Supplementary Materials — Supplementary Table 1: totality data vs sample data. Supplementary Table 2: best-worst case analysis. [file 6842825.f1.docx]

**Supporting Table1 Population data vs sample data**

| Clinical characteristics | Totality(5421) | Sample(4639) | p-value | 95%CI |
| --- | --- | --- | --- | --- |
| Age(years) | 58.11±8.64 | 58.07±8.64 | 0.76 | -0.30;0.22 |
| Male(%) | 33.6% | 33.5% | 0.91 |  |
| SBP(mmHg) | 163.22±24.21 | 163.31±24.19 | 0.799 | -0.61;0.79 |
| DBP(mmHg) | 96.90±12.66 | 96.99±12.64 | 0.624 | -0.27;0.46 |
| HR | 72.74±12.28 | 72.71±12.29 | 0.881 | -0.39;0.33 |
| GLU（mmol/L） | 5.57±1.69 | 5.57±1.69 | 0.948 | -0.051;0.047 |
| TC（mmol/L） | 5.53±1.10 | 5.53±1.10 | 0.647 | -0.024;0.040 |
| TG (mmol/L) | 1.68±1.23 | 1.69±1.24 | 0.753 | -0.031;0.042 |
| HDL-C（mmol/L） | 1.55±0.34 | 1.55±0.34 | 0.865 | -0.0092;0.01 |
| LDL-C（mmol/L） | 3.15±0.86 | 3.15±0.85 | 0.860 | -0.227;0.027 |
| BUN(mmol/L) | 5.47±1.81 | 5.47±1.81 | 0.885 | -0.057;0.049 |

**Supporting Table2 Best-Worst case analysis**

| index | Q1 | P-value | | Q2 | P-value | Q3 | P-value | | Q4 | P-value | |
| --- | --- | --- | --- | --- | --- | --- | --- | --- | --- | --- | --- |
| **BMI** |  | | | | | | | | | | |
| Best | Reference |  | 1.61(1.37;1.89) | | ＜0.01 | 2.29(1.95;2.68) | | ＜0.01 | 10.80(9.02;12.93) | | ＜0.01 |
| Worst | Reference |  | 1.61(1.37;1.89) | | ＜0.01 | 2.29(1.95;2.68) | | ＜0.01 | 1.03(0.87;1.21) | | 0.71 |
| **WC** |  | | | | | | | | | | |
| Best | Reference |  | 1.39(1.19;1.62) | | ＜0.01 | 1.58(1.35;1.85) | | ＜0.01 | 6.97(5.87;8.27) | | ＜0.01 |
| Worst | Reference |  | 1.39(1.19;1.62) | | ＜0.01 | 1.58(1.35;1.85) | | ＜0.01 | 0.63(0.53;0.75) | | ＜0.01 |
| **WHtR** |  | | | | | | | | | | |
| Best | Reference |  | 1.55(1.32;1.82) | | ＜0.01 | 2.20(1.87;2.57) | | ＜0.01 | 10.07(8.42;12.03) | | ＜0.01 |
| Worst | Reference |  | 1.55(1.32;1.82) | | ＜0.01 | 2.20(1.87;2.57) | | ＜0.01 | 0.97(0.82;1.14) | | 0.69 |
| **WHR** |  | | | | | | | | | | |
| Best | Reference |  | 1.19(1.02;1.38) | | ＜0.05 | 1.20(1.03;1.40) | | ＜0.05 | 4.65(3.95;5.47) | | ＜0.01 |
| Worst | Reference |  | 1.19(1.02;1.38) | | ＜0.05 | 1.20(1.03;1.40) | | ＜0.05 | 0.46(0.39;0.54) | | <0.01 |
